# Estimating the rate of retinal ganglion cell loss to detect glaucoma progression: An observational cohort study

**DOI:** 10.1097/MD.0000000000004209

**Published:** 2016-07-29

**Authors:** Kazuyuki Hirooka, Saeko Izumibata, Kaori Ukegawa, Eri Nitta, Akitaka Tsujikawa

**Affiliations:** Department of Ophthalmology, Kagawa University Faculty of Medicine, Miki, Kagawa, Japan.

**Keywords:** glaucoma progression, optical coherence tomography, retinal ganglion cell, visual field

## Abstract

This study aimed to evaluate the relationship between glaucoma progression and estimates of the retinal ganglion cells (RGCs) obtained by combining structural and functional measurements in patients with glaucoma.

In the present observational cohort study, we examined 116 eyes of 62 glaucoma patients. Using Cirrus optical coherence tomography (OCT), a minimum of 5 serial retinal nerve fiber layer (RNFL) measurements were performed in all eyes. There was a 3-year separation between the first and last measurements. Visual field (VF) testing was performed on the same day as the RNFL imaging using the Swedish Interactive Threshold Algorithm Standard 30–2 program of the Humphrey Field Analyzer. Estimates of the RGC counts were obtained from standard automated perimetry (SAP) and OCT, with a weighted average then used to determine a final estimate of the number of RGCs for each eye. Linear regression was used to calculate the rate of the RGC loss, and trend analysis was used to evaluate both serial RNFL thicknesses and VF progression.

Use of the average RNFL thickness parameter of OCT led to detection of progression in 14 of 116 eyes examined, whereas the mean deviation slope detected progression in 31 eyes. When the rates of RGC loss were used, progression was detected in 41 of the 116 eyes, with a mean rate of RGC loss of −28,260 ± 8110 cells/year.

Estimation of the rate of RGC loss by combining structural and functional measurements resulted in better detection of glaucoma progression compared to either OCT or SAP.

## 1 Introduction

Progressive optic neuropathy or glaucoma is characterized by the loss of both retinal ganglion cells (RGCs) and the retinal nerve fiber layer (RNFL), and leads to an associated visual field (VF) loss.^[1,2]^ Clinical practices traditionally assess the visual function by using standard automated perimetry (SAP). To prevent irreversible loss of the visual function once glaucoma progression is confirmed, treatments need to be modified or enhanced. Spectral-domain optical coherence tomography (SD-OCT) studies have specifically focused on the relationship between the structural and functional damage as a way of improving the detection of the progression of glaucomatous damage.^[3–6]^ Unfortunately, as reported in several studies, there has been poor agreement between the structure- and function-based results and the extent of the progression.^[3–5]^ Even so, the previous results overall have suggested that the SAP appears relatively insensitive when detecting changes during the early stage of glaucoma, whereas the OCT structural assessments appear to be relatively worse during the advanced stages of glaucoma.

Although it has not been possible to directly count the RGCs in vivo, clinical perimetry estimates of the RGC losses have been very close to the estimations of the RGC losses obtained during OCT assessments of the RNFL.^[7]^ A recent study by Medeiros et al^[8]^ reported that after combining structural and functional parameters, they were able to successfully estimate the RGCs. Moreover, the authors found that combining the structural and functional measurements to estimate the RGC loss resulted in a better detection of the progressive glaucomatous damage versus when using isolated structural and functional measurements. However, it should be noted that the previous study used the time-domain version of the OCT methodology to determine these measurements. A different study that did compare the SD-OCT to the time-domain OCT demonstrated that the SD-OCT was faster and provided more reproducible scans.^[9]^

Based on these previous findings, we designed the present study to evaluate whether combining the SD-OCT structure and function measurements to determine the RGC loss would provide a better way to detect the progressive glaucomatous damage.

## 2 Materials and methods

### 2.1 Patients

This retrospective review was performed at Kagawa University Hospital, Kagawa, Japan, and examined glaucoma patients who underwent OCT measurements of RNFL thickness and VF testing between October 2008 and March 2015. In accordance with the principles embodied in the Declaration of Helsinki, each eligible subject received a thorough explanation of the research protocol and signed an informed consent form. The Institutional Review Board at Kagawa University Hospital approved this study. Each subject underwent baseline ophthalmic evaluations including intraocular pressure testing, dilated fundus examination with stereoscopic biomicroscopy of the optic nerve head using slit-lamp and indirect ophthalmoscopy, and visual acuity testing with refraction. The inclusion and exclusion criteria and definitions used in the present study are based on those used in a previous study.^[10]^ The criteria for inclusion in this study were having a best-corrected visual acuity of 20/40 or better, a spherical error within a range between +4.0 and −6.0 diopters, and a cylinder within ±2.0 diopters. Subjects were excluded if they had any history of any kind of neurologic disease, retinal laser procedure, retinal pathology, or retinal surgery. Eyes that exhibited structural glaucomatous changes (vertical cup-disc asymmetry between fellow eyes of ≥0.2, a cup-disc ratio of ≥0.6, and a neuroretinal rim narrowing, notches, localized pallor, or RNFL defects with glaucomatous VF loss in the corresponding hemifield) were defined as glaucomatous eyes. To be defined as a glaucomatous VF, the glaucoma hemifield test was required to be outside of the normal limits on at least 2 consecutive baseline tests. Moreover, there needed to be at least 3 contiguous test points within the same hemifield on the pattern deviation plot at *P* < 1%, and at least one at *P* < 0.5%. This excluded any points on the edge of the field or those directly above and below the blind spot. VF and OCT testing of all eyes was conducted at approximately annual intervals. During the follow-ups, patients were required to have a minimum of 5 VF tests and 5 RNFL images to be included in the overall analysis. Both VF testing and RNFL imaging were performed during the same visit.

### 2.2 Cirrus HD-OCT RNFL measurement

Cirrus HD-OCT incorporates spectral domain technology (Carl Zeiss Meditec, Dublin, CA). After creating an optic disc cube from 3-dimensional data, Cirrus HD-OCT measurements can be extracted.^[6]^ From the 200 B-scans obtained, 200 A-scans over a 6-mm^2^ area at the center of the optic disc are selected for inclusion in the dataset. The RNFL thickness measurement and analysis methods have been described in detail elsewhere.^[6]^ After an RNFL thickness map is initially created based on the cube, the center of the disc is identified using software. Subsequently, the software extracts a 1.73-mm-radius circumpapillary circle from the dataset. For all images, it was necessary to have a signal strength >6 in order to be used in the present analysis. The OCT instrument calculated the RNFL thickness deviation and the RNFL thickness change maps; these data were exported to a computer for analysis of the progression pattern of the RNFL defects. For visualization of the RNFL defects, a 50 × 50 pixel RNFL thickness deviation map was generated. RNFL measurements below the 95% of the percentile ranges for that particular pixel were coded in yellow, whereas those below 99% were coded in red.

One of the components of the Guided Progression Analysis (GPA) software (Carl Zeiss Meditec Inc, Dublin, CA) is the RNFL thickness change map. Based on serial RNFL thickness maps, the software provides both event- and trend-based analysis of the RNFL progression. Furthermore, as the program can automatically align and register the baseline and follow-up OCT images, this ensures that changes can be measured at the same pixel locations. However, at least 4 patient visits are required to generate a GPA report. After obtaining and analyzing the images, the GPA program overlays and the compares the serial RNFL thickness versus the follow-up duration. The RNFL thickness progression was also assessed by the trend analysis function of the GPA software.

### 2.3 VF examination

To test for VF loss, we performed standard VF testing via the 30–2 Swedish Interactive Threshold Algorithm Standard test using static automated white-on-white threshold perimetry (Humphrey Field Analyzer II [HFA]; Carl Zeiss Meditec). HfaFiles version 5, which is part of the analytical program of the HFA, was used to evaluate the significance of the change over time. Using the VFs obtained during the follow-up period, the program performs a liner regression analysis of the mean deviation (MD) of the VFs, and then uses the results to calculate both a slope for the MD change per year and the statistical significance of this slope. Values of *P* < 0.05 for the change in the MD per year and MD slope were considered to indicate a significant change in the VF over time. For the VF to be defined as reliable, the fixation losses and the false-positive and false-negative rates were required to be <20%. Our study only included reliable test data in our analyses.

### 2.4 Combined structure and function estimate of retinal ganglion cell counts

A previous study has reported on the use of combined structure and function measurements to estimate the RGC counts.^[8]^ Briefly, after creating and using the formulas listed below, it was possible to estimate the number of RGC somas within a retinal area. This area corresponds to the specific SAP test field location at an eccentricity (*ec*) with a sensitivity (*s*) in decibels. The formulas are as follows:. 
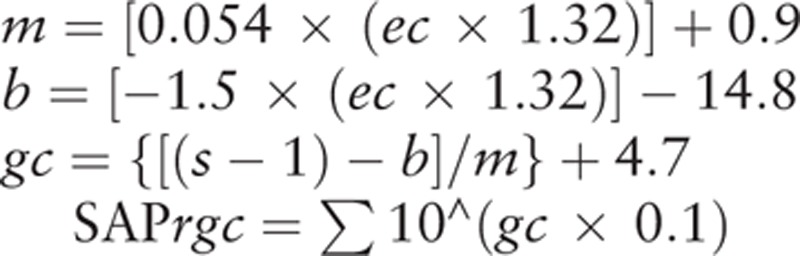


At a given *ec*, *m* and *b* will represent the respective slope and intercept of the linear function that describes the ganglion cell quantity (*gc*) in decibels to the VF *s* in decibels. For the perimetry measurements, each of the derived cell densities was considered to be uniform over the area of the retina that corresponded to the 6 × 6 degree area of visual space separating the test locations in the SAP. This made it possible to account for the total number of ganglion cells in an area of the retina. After adding all of the estimates from all of the locations in the VF, the SAP-derived estimate of the total number of RGCs (SAP*rgc*) was obtained using the above formulas. The main part of this model was designed to determine the estimated number of RGC axons from the RNFL thickness measurements that are obtained by the OCT. Based on the global RNFL thickness measurements obtained by OCT (OCT*rgc*), the total number of RGC axons can be derived by using the following formulas:. 
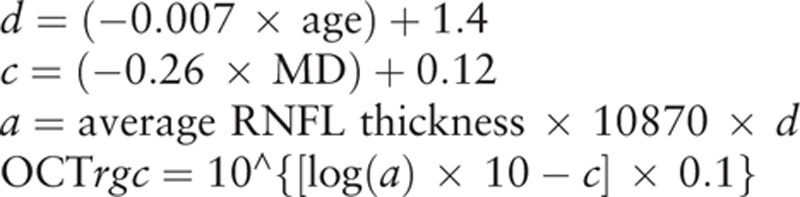


where d corresponds to the axonal density (axons/μm^2^), and c is a factor that corrects for the severity of the diseases and takes into account the remodeling of the RNFL axonal and nonaxonal composition. The OCT software automatically calculated the average RNFL thickness that corresponded to a 360-degree measurement area.

 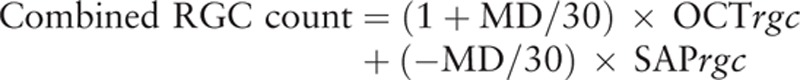


An inverse relationship exists between the disease severity and the estimates of the SAP and OCT. Thus, to reflect this inverse relationship, weights were chosen throughout the range of the normally reliable MD values, which ranged between 0 and −30 dB.

When the rate of RGC loss was found to be greater than the expected age-related loss, glaucomatous progression was considered to be present. A study by Kerrigan-Baumrind et al^[11]^ previously reported this value to be 7205 cells/year.

### 2.5 Analysis

The presence of a statistically significant negative slope was used to define the amount of progression that was shown by the OCT average thickness and MD slope. A Student *t* test was used to evaluate the differences between the progressive and nonprogressive eyes. All of the statistical values are presented as the mean ± standard deviation (SD), with *P* values <0.05 considered to indicate statistical significance. All statistical analyses were performed using SPSS version 19.0 (IBM, New York, NY).

## 3 Results

Table 1 presents the results of the 694 OCT scans and 694 VF tests that were analyzed. The scans were obtained from 116 eyes of 62 glaucoma patients, with an average number of 6.0 OCT scans and VF tests performed in each eye. The follow-up duration ranged from 48 to 72 months. Table 1 also presents both the patient's demographics, and the VF and RNFL measurements. Mean age was 61.7 ± 10.7 years. Baseline VF MD and RNFL thickness values were −6.42 ± 6.14 dB (range 1.08 to −27.63 dB) and 69.8 ± 11.6 μm (range 47–104 μm), respectively. At baseline, the mean number of RGCs was 576,786 ± 211,467 cells (range 108,692 to 1,063,009 cells).

**Table 1:**
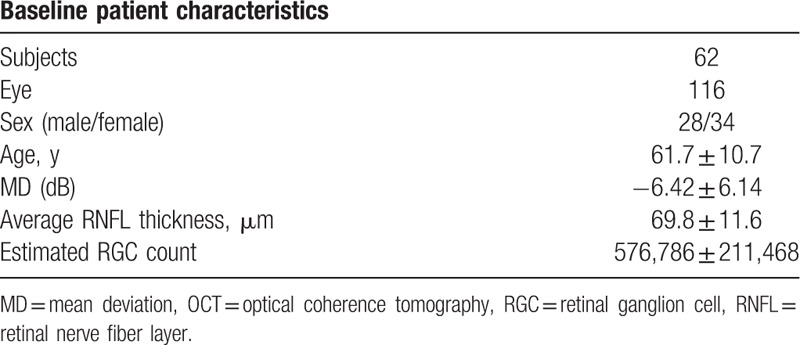
Demographics, visual field, and OCT RNFL measurements.

**Figure 1:**
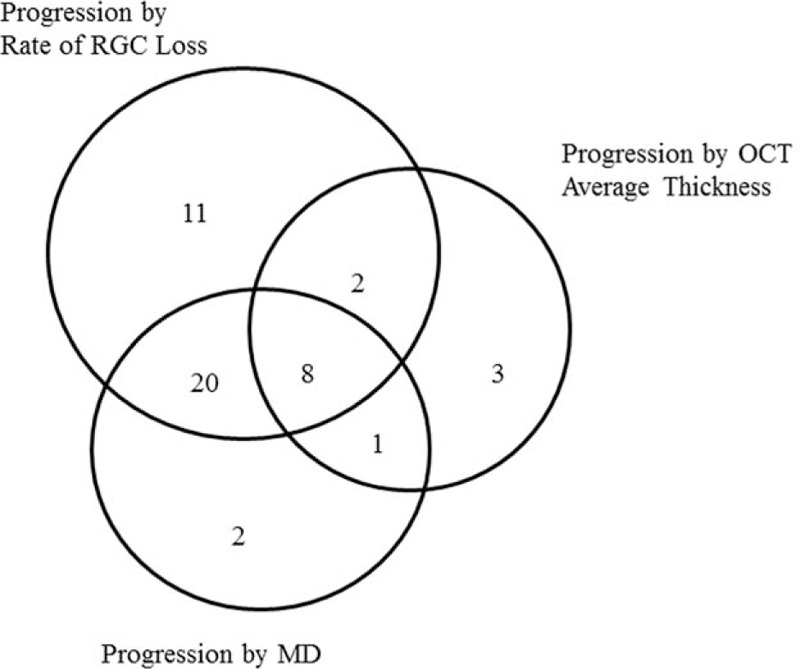
A Venn diagram comparing the number of eyes detected as progression according to the rate of RGC loss, OCT average thickness parameter, and HFA MD slope. The number of subjects is shown in brackets. HFA = Humphrey Field Analyzer II, MD = mean deviation, OCT = optical coherence tomography, RGC = retinal ganglion cell.

Figure 1 shows the proportional Venn diagram for the number of eyes with progression that were detected by each method. Using the average RNFL thickness parameter of the OCT, we were able to detect progression in 14 of the 116 eyes. When using the MD slope, progression was detected in 31 eyes. A total of 41 eyes exhibited a statistically significant rate of RGC loss, with a mean rate of RGC loss of −28,260 ± 12,268 cells/year (range −12,389 to −58,123 cells/year) (Table 2). There were 13 eyes in which progression was detected by the RGC loss but not by the MD slope. In addition, the MD slope detected progression in 3 eyes for which the RGC loss indicated no progression. Comparison of these 2 groups revealed that when progression was found based on only the rate of RGC loss, these eyes appeared to exhibit faster structural change rates versus the eyes shown to have progression by only the MD slope as determined by the OCT measurements of the average RNFL thickness (−0.59 ± 0.56 μm/year versus 0.66 ± 1.94 μm/year, respectively; *P* = 0.04). In 31 eyes, progression was detected by the rate of RGC loss, but not by the OCT average thickness. However, in 4 of the eyes, progression was detected by the OCT average thickness, but not by the rate of RGC loss. The mean rate of RGC loss was −26,706 ± 10,916 cells/year in the former versus −14,703 ± 18,845 cells/year in the latter group. Moreover, when measurements were made using the MD slope, eyes found to have progression based on only the rate of RGC loss exhibited significantly faster rates of functional change than eyes reported to have progression based on only OCT (−0.72 ± 0.48 dB/year versus -0.06 ± 0.45 dB/year, respectively; *P* = 0.01).

**Table 2:**
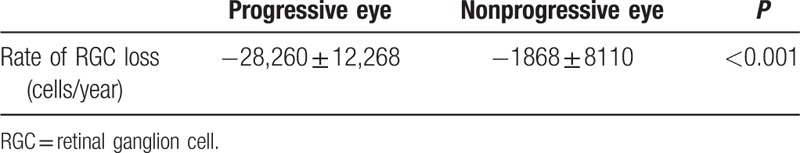
Mean rate of RGC loss.

## 4 Discussion

Progression of glaucoma can be determined based on structural and/or functional changes. When progression of glaucoma is confirmed in patients, modification or enhancements of the treatments is necessary to prevent further irreversible loss of the visual function. In the present study, we examined the use of combining structure and function measurements in glaucoma patients to determine the relationship between glaucoma progression and the estimates of the RGC counts.

Previous studies have recommended both VF and digital imaging devices be used to monitor glaucoma patients.^[12]^ However, there has been considerable variation in the results when different structural and functional tests have been used to detect disease progression.^[3–5]^ In our own recent study, we reported finding a good agreement between the structural and functional tests that were used to evaluate glaucoma progression during the early stages of the disease.^[13]^ In addition, it has also been reported that when OCT was used during advanced stages of the glaucomatous damage, the structural assessments were much worse.^[14]^ The results of all of these previous studies suggest that when detecting changes in early glaucoma, HFA appears to be relatively insensitive, whereas OCT assessments are relatively worse during advanced stages of glaucoma. Therefore, overall these findings indicate the importance of using a combined approach when detecting glaucoma progression.

In the present study, we used both HFA and OCT to estimate the RGC losses. As a result, this made it possible to detect the majority of eyes that had progression, regardless of the glaucoma stage. Even so, as seen in Figure 1, SAP and OCT were only able to detect progression in 1 eye, with no detection found in any of the eyes when only using the calculated rate of the RGC loss. In the 1 eye in which progression was detected, the rate of RGC loss was −14,935 cells/year, whereas the rates for the MD and OCT thickness changes were −0.53 dB/year and −1.05 μm/year, respectively. In contrast, when we examined the progression based on the rate of the RGC loss, we were able to detect progression in 11 eyes. Neither MD nor OCT was able to detect progression in any of these same eyes. The mean rate of the RGC loss in these 11 eyes was −24,764 cells/year, whereas the mean rates for the MD and OCT average thickness changes were −0.50 dB/year and −0.05 μm/year, respectively. Overall, the number of eyes found to have progressed was higher when based on the rates of RGC loss versus when a simple linear regression analysis of the MD was performed.

Medeiros et al^[8]^ previously reported achieving better results for estimating the rate of RGC loss and detecting progressive glaucomatous damage when the structure and function measurements were combined versus when using isolated structure and function measurements. However, it should be noted that there were 3 distinct differences between this previous report and our present study. The first difference was related to the type of device used. In a previous study, we compared measurements between the Stratus and Cirrus devices. Our results indicated that when a thinner RNFL thickness was present, the Stratus measurements tended to be thinner than those by the Cirrus, whereas when a thicker RNFL thickness was present, the Stratus measurements tended to be thicker than those by the Cirrus.^[15]^ Furthermore, better RNFL measurement reproducibility has been reported for the SD-OCT versus the time-domain OCT.^[9]^ The second difference between the 2 studies concerned the determination of the rates for the VF loss. In the Medeiros et al's^[16]^ study, they used the VF index (VFI) to evaluate the rate of the VF loss. However, it has been shown that both the VFI and MD can be used to measure the glaucoma progression, as they are equally effective at all stages of the disease. Therefore, our study used the MD instead of the VFI to determine the progression.

There were some limitations for our present work, which included having only a small sample size and a small number of tests. This can be easily rectified by undertaking larger studies that should be able to more precisely detect the glaucoma progression when using this combined structure and function approach. Another possible limitation of our study is that when we estimated the number of RGCs by combining the SAP and OCT data, we used the same empirical formulas reported by Medeiros et al.^[8]^ Thus, it should be noted that this validation was performed in monkeys and was not based on direct histologic RGC counts in humans.

Although the use of RGC loss was able to detect glaucoma progression in many eyes, we were also able to detect progression in 27 eyes when using both SAP and OCT. Even so, estimations of the rate of the RGC loss based on the combined structure and function measurements performed much better overall than either OCT or SAP when detecting the progression of glaucoma.
